# Assessing fetal growth impairments based on family data as a tool for identifying high-risk babies. An example with neonatal mortality

**DOI:** 10.1186/1471-2393-7-28

**Published:** 2007-11-28

**Authors:** Carsten B Pedersen, Yuelian Sun, Mogens Vestergaard, Jørn Olsen, Olga Basso

**Affiliations:** 1 National Centre for Register-based Research, University of Aarhus, Aarhus, Denmark; 2 Institute of Public Health, Department of Epidemiology, University of Aarhus, Aarhus, Denmark; 3 Institute of Public Health, Department of General Practice, University of Aarhus, Aarhus, Denmark; 4 Department of Epidemiology, School of Public Health, UCLA, Los Angeles, CA, USA; 5 Epidemiology Branch, National Institutes of Environmental Health Sciences (National Institutes of Health, Department of Health and Human Services), Research Triangle Park, NC, USA

## Abstract

**Background:**

Low birth weight is associated with an increased risk of neonatal and infant mortality and morbidity, as well as with other adverse conditions later in life. Since the birth weight-specific mortality of a second child depends on the birth weight of an older sibling, a failure to achieve the biologically intended size appears to increase the risk of adverse outcome even in babies who are not classified as small for gestation. In this study, we aimed at quantifying the risk of neonatal death as a function of a baby's failure to fulfil its biologic growth potential across the whole distribution of birth weight.

**Methods:**

We predicted the birth weight of 411,957 second babies born in Denmark (1979–2002), given the birth weight of the first, and examined how the ratio of achieved birth weight to predicted birth weight performed in predicting neonatal mortality.

**Results:**

For any achieved birth weight category, the risk of neonatal death increased with decreasing birth weight ratio. However, the risk of neonatal death increased with decreasing birth weight, even among babies who achieved their predicted birth weight.

**Conclusion:**

While a low achieved birth weight was a stronger predictor of mortality, a failure to achieve the predicted birth weight was associated with increased mortality at virtually all birth weights. Use of family data may allow identification of children at risk of adverse health outcomes, especially among babies with apparently "normal" growth.

## Background

Birth weight correlates with the risk of perinatal and infant mortality and morbidity [1-4], as well as with a number of health conditions later in life, including cardiovascular diseases, type 2 diabetes, obesity [5-7], and cognitive function [8]. However, not all small babies are growth-restricted, as some will just be constitutionally small [9], and not all babies of "normal" size are appropriately grown. For this reason, we need better methods to identify growth restricted babies, especially in epidemiological studies. There is a strong tendency to repeat birth weight in successive births of the same mother [10-13], and several studies have shown that mortality in second babies varies not only as a function of their own birth weight, but depends also on the birth weight of their older sibling, as small babies whose older sibling was also small had lower mortality than small babies whose older sibling was large [10,11,14,15]. These studies, however, did not take gestational age of either child into consideration. Basso et al [16] showed that classifying babies as "growth restricted" based on their expected size predicted delayed motor development at 6 months slightly better than the usual criterion of small-for-gestation, and that combining the two criteria may thus improve prediction. Mortality is recorded essentially without error and could be considered as an extreme on a continuum of unfavourable outcomes. Quantifying the risk associated with the deviation from one's predicted birth weight, regardless of the birth weight itself, will strengthen the case for early identification of at-risk babies, especially in circumstances where there is no obvious fetal growth restriction.

In this study, we aimed at quantifying the risk of neonatal death as a function of a baby's failure to fulfil its predicted growth potential across the whole distribution of birth weight, including the "normal" range. To this end, we predicted the birth weight of 411,957 second babies born alive in Denmark between 1979 and 2002 using a modified version of the method proposed by Skjaerven et al [12].

## Methods

### Data

The Danish Civil Registration System (CRS) [17], which includes continuously updated information on vital status, was established in 1968, when all residents of Denmark were assigned a unique identifier (CRS number). All Danish national registries are based on this identifier, enabling accurate linkage between them. We linked the individual information recorded in the CRS to that of the Danish Medical Birth Registry [18] which includes the birth record of all live births in Denmark since 1973. Due to secular changes in recording, we restricted our analysis to children born between 1979 and 2002, the most recent year with fully updated information at the time this study was initiated. Because of digit preference primarily to the nearest 100 grams, we rounded birth weight to the nearest 100 grams interval. Gestational age is based on the date of last menstrual period, but often corrected by ultrasound measurements, especially in the most recent period. Gestational age was recorded in completed weeks between 1978 and 1996, and in days from 1997 onward.

During the study period, there were 1,476,753 live births to Danish residents, all of which are included in the Medical Birth Registry. We excluded 51,372 (3.5%) births due to missing information on birth weight or gestational age.

### Inconsistencies in gestational age

Gestational age is often estimated with error [19-24], with an excess of unlikely large birth weights among infants with a low gestational age. To assess the consistency between birth weight and gestational age among preterm births, we applied a strategy similar to the mixture of two Normal distributions used previously [20,23]. Maximum likelihood estimates of the mixture model parameters were obtained using the Newton-Rapson Method [25]. We stratified the data by sex and gestational age (Table 1). Among very preterm births (22–33 weeks), we observed a bimodal distribution and thus considered the data as inconsistent if the observed birth weight was greater than three times the standard deviation of the major Gaussian component of the distribution. Overall, 629 (2.7%) of the 23,425 very preterm births were considered inconsistent (see Table 1). We also applied the mixture model to gestational ages beyond 33 weeks. Like Tentoni et al [20], we found that the two model components overlapped almost completely, thus making the correction unnecessary.

**Table 1:** Parameters of the major and secondary components in the mixture of two Normal distributions of birth weight among preterm births (22–33 weeks) stratified by gestational age and sex

Gestational age (weeks)	22–23	24–25	26–27	28	29	30	31	32	33
**Girls**									
Sample size	49	93	507	2017	917	1464	1721	2659	3503
μ_m_: Mean major Gaussian (g)	556	713	913	1071	1216	1344	1541	1730	1934
σ_m_: Std major Gaussian (g)	125	134	182	213	258	277	306	317	363
μ_s_: Mean sec. Gaussian (g)	2771	3800	2141	2848	2182	3103	2556	2207	2322
σ_s_: Std sec. Gaussian (g)	591	150	805	657	532	613	1244	787	755
p_s_: Weight sec. Gaussian (%)	3.6	0.5	3.3	2.7	1.9	8.2	4.5	7.8	9.8
Threshold (g) &	931	1115	1459	1710	1990	2175	2459	2681	3023
Number of births above threshold	5	5	25	20	17	92	35	52	51
									
**Boys**									
Sample size	49	85	422	1629	808	1176	1357	2111	2956
μ_m_: Mean major Gaussian (g)	601	769	969	1148	1299	1434	1629	1828	2040
σ_m_: Std major Gaussian (g)	119	150	191	224	243	298	322	335	378
μ_s_: Mean sec. Gaussian (g)	2850	3479	2706	2662	2209	3301	2808	2248	3331
σ_s_: Std sec. Gaussian (g)	354	991	947	978	1105	907	1433	842	551
p_s_: Weight sec. Gaussian (%)	4.3	2.7	2.3	3.3	4.9	7.8	3.2	7.2	1.4
Threshold (g) &	958	1219	1542	1820	2028	2328	2595	2833	3174
Number of births above threshold	4	16	28	28	27	103	34	54	33

& The threshold was estimated as the mean μ_m _of the major Gaussian distribution plus 3 times the standard deviation σ_m _of the major Gaussian distribution. Birth weights above this threshold were considered as inconsistent with gestational age and excluded from the analysis.

### Identification of sibships

Using data on family members recorded in the CRS [17], we identified all sibships in Denmark consisting of first- and second-born singletons born alive between 1979 and 2002 (481,526 sibships). The pair of siblings had the same mother, but not necessarily the same father. We restricted the analyses to sibships with available and credible information on birth weight and gestational age of both babies, and to instances in which both live-born babies had a gestational age of at least 28 completed weeks (411,957 sibships). The included sibships constitute 86% (= 411,957/481,526) of the total number of sibships where the first and second baby were both born in Denmark during the study period.

### Prediction of birth weight in second-born babies

We first calculated the predicted birth weight of second-born babies using the strategy described by Skjaerven et al [12], based on the younger sibling's sex and gestational age and on the older sibling's birth weight. Since this approach did not take into consideration either the first-born's sex or gestational age, we explored whether including these factors improved the prediction. Since the variance of birth weight increases with increasing gestational age, the model used to predict the birth weight of the second-born needs to allow for this heterogeneity. Therefore, we used a variance component model [26] to predict the second-born's birth weight, using the first birth weight as a linear term for each stratum of gestational age of first- and second-born babies, with a common sex correction. The birth weights of the second-born babies were assumed to be independent and normally distributed with a variance depending on their gestational age only. The model used to predict the absolute birth weight of the second-born baby was analogous to using a separate linear normal regressions for each stratum of gestational age of first- and second-born babies, except that we used a common sex correction independent of the gestational age of either baby and we constrained the variance of the second-born babies birth weights to be the same across all first-born's gestational ages.

We thus used the following equation to predict the birth weight of the second-born baby:

**Pred**(**W**_**2**_) = **I**(**g**_**1**_, **g**_**2**_) + **β**(**g**_**1**_, **g**_**2**_) * **w**_**1 **_+ **γ**(**s**_**1**_, **s**_**2**_),

where

**Pred**(**W**_**2**_): predicted birth weight of the second-born child,

**I**(**g**_**1**_, **g**_**2**_): estimated intercept among sibs where the first-born had a gestational age of **g**_**1 **_and the second-born had a gestational age of **g**_**2**_.

**β**(**g**_**1**_, **g**_**2**_): estimated slope among sibs where the first-born had a gestational age of **g**_**1 **_and the second-born had a gestational age of **g**_**2**_

**w**_**1**_: first-born baby's observed birth weight

**γ**(**s**_**1**_, **s**_**2**_): estimated sex correction depending of the first-borns sex (**s**_**1**_) and the second-borns sex (**s**_**2**_)

Note: All parameters were estimates simultaneously using a variance component model.

In these analyses, first-borns' gestational age (**g**_**1**_) was categorized as 28–33, 34–35, 36–37, 38–39, 40–41, and >= 42 completed weeks, and second-borns' gestational age (**g**_**2**_) was categorized as 28–29, 30–31, 32–33, 34, 35, 36, 37, 38, 39, 40, 41, and >= 42 weeks. Since we expected gestational age of the second-born babies to be more important for predicting their own birth weight than gestational age of their older sibling, we decided a priori to use a more detailed categorization of gestational age for the second-born. For each gestational age of the first- and second-born, the parameters of intercept **I**(**g**_**1**_, **g**_**2**_) and slope **β**(**g**_**1**_, **g**_**2**_) are presented in Table 2. At the foot of the Table, the estimated common sex correction **γ**(**s**_**1**_, **s**_**2**_) is shown for each of the four combinations (female – female, female – male, male – female, male – male).

**Table 2:** Parameters of intercept (I) and slope (β) for predicting the birth weights of second-born children based on gestational age and sex * of first- and second-born children.

Gestational age of second child (g_1_: weeks)	Gestational age of first child (g_2_: weeks)																		
		
	28–33			34–35			36–37			38–39			40–41			>= 42			SD
		
	No.	I	β	No.	I	β	No.	I	β	No.	I	β	No.	I	β	No.	I	β	
28–29	62	900	0.198	46	615	0.283	55	850	0.150	164	1030	0.066	190	735	0.148	37	840	0.111	240
30–31	117	980	0.348	65	740	0.385	114	1110	0.177	256	1050	0.168	309	880	0.207	42	195	0.359	320
32–33	167	1430	0.360	149	1270	0.312	293	920	0.407	557	1470	0.171	613	1230	0.224	78	1220	0.231	360
34	144	1650	0.407	196	1395	0.450	281	1075	0.462	563	1150	0.382	591	1140	0.352	83	1265	0.304	440
35	200	1675	0.496	261	1285	0.575	529	865	0.632	925	1095	0.481	941	1230	0.397	124	1255	0.379	440
36	301	2005	0.439	426	1140	0.722	1107	1110	0.649	2026	1335	0.490	1975	1365	0.439	244	1500	0.378	435
37	495	2450	0.341	712	1705	0.566	2420	1465	0.588	5388	1380	0.553	5147	1270	0.539	619	1385	0.485	435
38	659	2660	0.296	1132	2095	0.488	4263	1735	0.546	16229	1650	0.526	17312	1560	0.521	2329	1685	0.468	400
39	806	3050	0.212	1385	2355	0.448	5872	2030	0.501	31014	1750	0.541	43119	1690	0.534	5487	1790	0.490	385
40–41	1204	3295	0.171	1863	2785	0.344	8110	2345	0.446	51057	1855	0.553	132010	1780	0.554	25393	1905	0.510	390
>= 42	145	3520	0.125	182	3385	0.184	695	2670	0.382	4692	2220	0.488	19508	1855	0.568	8479	1960	0.531	415

No: Number of sibships included.

SD: Estimated Standard Deviation based on the regression model.

* The estimated sex correction (**γ(s**_1_, **s**_2_)) is:

-- 1.st female – 2.nd female: -65 grams

-- 1.st female – 2.nd male: +85 grams,

-- 1.st male – 2.nd female: -145 grams,

-- 1.st male – 2.nd male: 0 grams (reference).

Example

According to the model used, for a baby boy at 39 weeks of gestation with an older brother who weighed 2000 grams at 34–35 week of gestation, had a predicted birth weight of 3251 grams (Table 2, **I**(**g**_**1 **_= **34–35**, **g**_**2 **_= **39**) = 2355, **β**(**g**_**1 **_= **34–35**, **g**_**2 **_= **39**) = 0.448, **γ**(**s**_**1 **_= **male**, **s**_**2 **_= **male**) = 0, i.e., predicted birth weight = 2355 + 0.448 * 2000 grams + 0). Similarly, if the second-born was a girl, she would have a predicted birth weight of 3106 grams (Table 2, **I**(**g**_**1 **_= **34–35**, **g**_**2 **_= **39**) = 2355, **β**(**g**_**1 **_= **34–35**, **g**_**2 **_= **39**) = 0.448, **γ**(**s**_**1 **_= **male**, **s**_**2 **_= **female**) = -145 grams, i.e., predicted birth weight = 2355 + 0.448 * 2000 grams – 145 grams).

Initially, we investigated the assumption that the variance of the second-born babies birth weights were the same across all first-born's gestational ages. Though, significant variation was observed, the estimated variance seemed to vary at no meaningful pattern, and we thus decided not to included this term in the model.

### Estimating the relative risk of neonatal death

Second-born babies were followed from birth to the 27^th ^day of life, death, or emigration from Denmark, whichever came first. Neonatal death was defined as death occurring within 27 days after birth. We estimated the relative risk of neonatal death as a function of the deviation from the predicted birth weight, expressed by the birth weight ratio [(observed birth weight)/(predicted birth weight) × 100]. The relative risk of death was estimated by log-linear Poisson regression treating the number of person-years as an offset variable [27,28]. All estimated relative risks were adjusted for year of birth, sex, and gestational age of the second baby. Though, we acknowledge that the reported relative risks are in principle incidence rate ratios, we prefer to refer to these as relative risks as most readers are familiar with this term.

## Results

### Prediction of birth weight using family data

**Figure 1:**
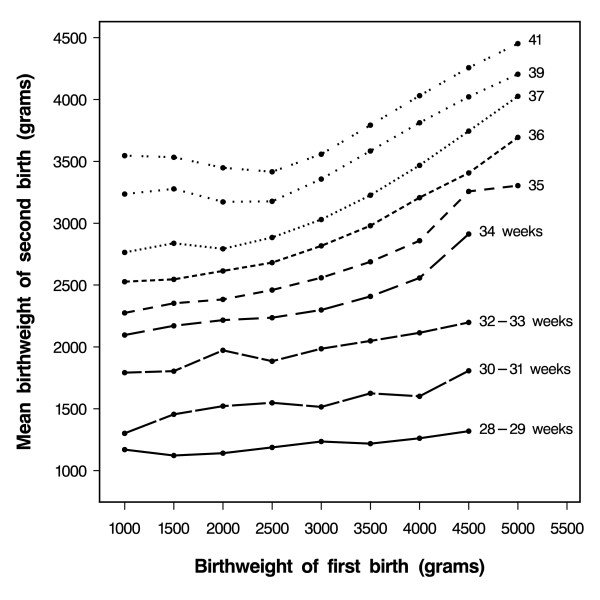
Gestational-age specific mean birth weight of second-born children according to the birth weight of their older siblings (Only mean values based on more than 10 babies are shown.)

Figure 1 shows the mean second-born's birth weight according their gestational age and birth weight of their older sibling. At the 39^th ^week of gestation, babies whose older sibling weighed 2000 grams had a mean birth weight of 3175 grams, while babies whose older sibling weighed 5000 grams had a mean birth weight of 4200 grams. These patterns were strikingly similar to those reported by Skjaerven et al [12], who found that, at any given gestational age of the second-born child, there was a linear association between its birth weight and that of the first-born. Additionally, they argued that this association was restricted to sibships in which the first-born weighed at least 2500 grams. Thus, they used a constant term to model the second-born's birth weight when the older sibling weighed less than 2500 grams.

**Figure 2:**
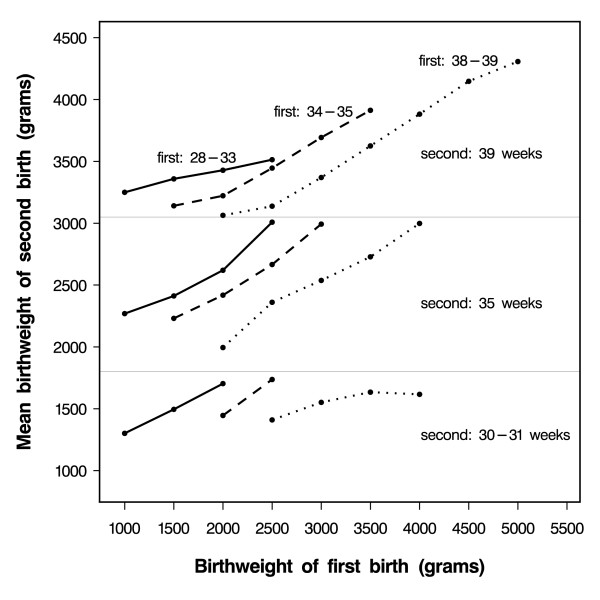
Mean birth weight of second-born children at selected gestational ages [39 (top), 35 (middle), or 30–31 (bottom) weeks] according to the birth weight of first-born children at selected gestational ages [28–33 (solid lines __), 34–35 (punctuated lines ---), and 38–39 (dashed lines ...) weeks]. (Only mean values based on more than 10 babies are shown.)

However, when we stratified the results shown in Figure 1 by the first-born's gestational age (Figure 2), we observed a linear association between the second-born's birth weight and the first-born's birth weight for all birth weights of the first-born, including birth weights below 2500 grams. Therefore, we modelled the second-born's birth weight, using the first-born's birth weight as a linear term for each stratum of gestational age of first- and second-born babies. Table 2 shows the estimated parameters obtained through this method, which we used to predict the birth weight of the second-born child.

To compare our prediction with that proposed by Skjaerven et al [12], we applied their equation to the Danish data (results not shown). The overall R-square estimated using our model was 0.48, very close to that based on the Norwegian model [12] which was 0.46. However, the R-square by the first-borns' gestational age showed that, the lower the gestational age of the first-born, the better the fit of our model compared to the Norwegian model.

### Estimation of the risk of neonatal mortality using family data

Among the 411,957 second-born children included in the study, 946 died within 27 days after birth, and 30 were lost to follow-up due to emigration from Denmark and were thus censored at the time of emigration.

Within each category of the achieved birth weight, the risk of neonatal death depended on the birth weight ratio (Table 3). In addition, within each category of birth weight ratio, the lower the achieved birth weight, the greater the absolute risk of neonatal death. This was also observed among babies who achieved their predicted birth weight defined as within 90–110% of the predicted birth weight.

**Table 3:** Number of neonatal deaths, number of babies, and absolute risk according to achieved birth weight and birth weight ratio & among 411957 (including 946 neonatal deaths) second-born babies (Denmark, 1979–2002)

Birth Weight Ratio &		Achieved birth weight							
		
		<1500 g	1500–1999 g	2000–2499 g	2500–2999 g	3000–3499 g	3500–3999 g	>= 4000 g	Total
< 50%	Deaths	60	14	0	0	0	0	0	74
	Babies	137	35	0	0	0	0	0	172
	Absolute risk †	437.96	400.00	-	-	-	-	-	430.23
50–60%	Deaths	22	20	8	0	0	0	0	50
	Babies	120	184	89	1	0	0	0	394
	Absolute risk †	183.33	108.70	89.89	-	-	-	-	126.90
60–70%	Deaths	15	9	24	6	0	0	0	54
	Babies	133	327	923	287	7	0	0	1677
	Absolute risk †	122.78	27.52	26.00	20.91	-	-	-	32.20
70–80%	Deaths	23	14	34	36	5	0	0	112
	Babies	205	374	2182	6585	1473	24	0	10843
	Absolute risk †	112.20	37.43	15.58	5.47	3.39	-	-	10.33
80–90%	Deaths	27	20	16	47	59	7	0	176
	Babies	203	460	1995	18662	32431	4355	58	58164
	Absolute risk †	133.01	43.48	8.02	2.52	1.82	1.61	-	3.03
90–110% *	Deaths	35	31	30	40	100	91	32	359
	Babies	368	779	2009	12331	88402	125576	39621	269086
	Absolute risk †	95.11	39.80	14.93	3.24	1.13	0.72	0.81	1.33
110–120%	Deaths	17	8	7	7	5	17	19	80
	Babies	90	139	351	706	3208	16228	34574	55296
	Absolute risk †	188.89	57.55	19.94	9.92	1.56	1.05	0.55	1.45
>= 120%	Deaths	5	6	6	6	4	5	9	41
	Babies	30	121	191	346	702	2268	12667	16325
	Absolute risk †	166.67	49.59	31.41	17.34	5.70	2.20	0.71	2.51

Total	Deaths	204	122	125	142	173	120	60	946
	Babies	1286	2419	7740	38918	126223	148451	86920	411957
	Absolute risk †	158.63	50.43	16.15	3.65	1.37	0.81	0.69	2.30

& Achieved birth weight divided by predicted birth weight times 100

† Crude absolute risk of neonatal death per 1000 babies.

* Babies who achieved their predicted birth weight within +/- 10%.

- indicates that the data were too sparse to provide a reliable estimate

**Figure 3:**
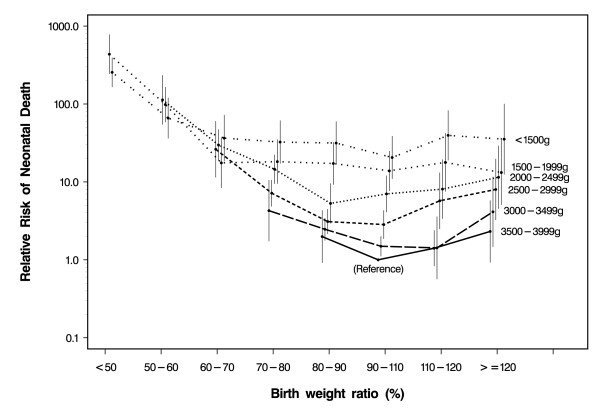
Adjusted relative risk of neonatal death (log scale) of second-born children according to the birth weight ratio by stratifying the achieved birth weight (Babies with an achieved birth weight of 3500–3999 g and a birth weight ratio of 90–109% were chosen as the reference). Estimates were based on 411957 babies, including 946 neonatal deaths (Denmark 1977–2000). Vertical bars indicate 95% confidence intervals.

Figure 3 shows the adjusted relative risks of neonatal death (in log scale) for second-born children according to the birth weight ratio and the achieved birth weight. Babies weighing 3500–3999 grams who had achieved their predicted birth weight were chosen as the reference category. The achieved birth weight was strongly predictive of neonatal death, although adjustment for gestational age attenuated the estimates. Among babies who achieved their predicted birth weight and using 3500–3999 grams as the reference category, the estimated relative risks were 20.6 (95% CI: 11.0–38.6) for babies weighing <1500 grams, 13.9 (95% CI:7.7–24.9) for those weighing 1500–1999 grams, 7.0 (95% CI: 4.1–12.0) for those with a birth weight between 2000 and 2499 grams, 2.8 (95% CI: 1.8–4.3) for those weighing 2500–2999 grams, and 1.5 (95% CI: 1.1–2.0) for those who weighed 3000–3499 grams. Among babies who weighed 3500–3999 grams, however, those who achieved 80–90% of the predicted birth weight had a relative risk of 2.0 (95% CI: 0.9–4.3) compared with those who achieved the predicted birth weight within ± 10%. Those who exceeded their predicted birth weight also had slightly elevated, although not statistically significant, relative risks [1.4 (95% CI: 0.8–2.4) among babies who achieved 110–120% of the predicted birth weight and 2.3 (95% CI: 0.9–5.8), among those who achieved more than 120% of their predicted birth weight].

Restricting the analysis to second-born babies born at term (37–41 weeks) whose older siblings were alive at their first birthday yielded similar results (Table 4), but the sample size was considerably reduced.

**Table 4:** Number of neonatal deaths, number of babies, and absolute risk according to achieved birth weight and birth weight ratio& among 360523 second-born term babies (37–41 weeks) whose older sibling were alive at the first birthday (504 neonatal deaths) (Denmark, 1979–2002).

Birth Weight Ratio &		Achieved birth weight							
		
		<1500 g	1500–1999 g	2000–2499 g	2500–2999 g	3000–3499 g	3500–3999 g	>= 4000 g	Total
< 50%	Deaths	37	14	0	0	0	0	0	51
	Babies	67	33	0	0	0	0	0	100
	Absolute risk †	552.24	424.24	-	-	-	-	-	510.00
50–60%	Deaths	1	13	8	0	0	0	0	22
	Babies	9	147	85	1	0	0	0	242
	Absolute risk †	-	88.44	94.12	-	-	-	-	90.91
60–70%	Deaths	0	1	21	5	0	0	0	27
	Babies	0	140	856	233	6	0	0	1235
	Absolute risk †	-	-	24.53	21.46	-	-	-	21.86
70–80%	Deaths	0	0	20	30	3	0	0	53
	Babies	0	44	1829	6047	1153	16	0	9098
	Absolute risk †	-	-	10.93	4.96	2.60	-	-	5.83
80–90%	Deaths	0	0	3	43	54	6	0	106
	Babies	0	6	935	17559	28852	3231	39	50622
	Absolute risk †	-	-	3.20	2.45	1.87	1.86	-	2.09
90–110% *	Deaths	0	0	1	12	88	76	27	204
	Babies	0	1	162	9092	83708	113119	31627	237709
	Absolute risk †	-	-	-	1.31	1.05	0.67	0.85	0.86
110–120%	Deaths	0	0	0	0	1	16	14	31
	Babies	0	0	2	116	2408	15527	30146	48199
	Absolute risk †	-	-	-	-	-	1.03	0.46	0.64
>= 120%	Deaths	0	0	0	0	1	1	8	10
	Babies	0	0	2	20	279	1842	11184	13327
	Absolute risk †	-	-	-	-	-	-	0.71	0.75

Total	Deaths	38	28	53	90	147	99	49	504
	Babies	76	371	3871	33068	116406	133735	72996	360523
	Absolute risk †	500.00	75.47	13.69	2.72	1.26	0.74	0.67	1.39

& Achieved birth weight divided by predicted birth weight times 100

† Crude absolute risk of neonatal death per 1000 babies.

* Babies who achieved their predicted birth weight within +/- 10%.

- indicates that the data were too sparse to provide a reliable estimate

In general, for any achieved birth weight above 2000 grams, there was an inverse J-shaped association between the birth weight ratio and the relative risk of neonatal death. Babies with the lowest birth weight ratio had the highest relative risk. Except for the category 2500–2999 grams, babies who achieved their predicted birth weight had the lowest risk. Adding the birth weight ratio to the model already including birth weight and gestational age significantly improved the fit (chi-square = 252.41, df = 32, p < 0.0001).

## Discussion

In this study, failure to fulfil the estimated growth potential increased the risk of neonatal death. Several authors [10,11,14,15] indicated that weight-specific mortality of the second child depends in part on the birth weight of the first child. After additionally taking into consideration gestational age of both infants, our observations further suggest that deviations from the predicted birth weight contribute to mortality across the whole distribution of birth weights. At virtually all birth weights, babies whose achieved birth weight was below 90% of the predicted had an increased risk of neonatal death. Thus, prediction of neonatal death in second-born babies is significantly improved by adding the deviation from the predicted birth weight; even though a low achieved birth weight remained the stronger predictor.

The heterogeneity in risk of neonatal death observed among babies within the same weight category provides further evidence that birth weight alone is an incomplete marker of fetal growth although, used alone, the birth weight ratio would be an even poorer predictor at the lower birth weights. However, in the absence of clinical information, the birth weight ratio may be particularly useful in the "normal" range of birth weight.

### Methodological considerations in predicting birth weight

Unlike Skjaerven et al [12], we chose not to adjust for trends in birth weight by calendar time, since an increase in birth weight with birth year (6 grams per year) was in part accounted for by the older sibling's birth weight, and since the interval between pregnancies was relatively short (mean: 3.5 yrs, SD ± 2.0 yrs). Additionally, we took into consideration sex and gestational age of both the first and second babies, rather than only those of the second, as proposed by Skjaerven et al [12]. However, to reduce the complexity of the prediction equation, our correction for sex was the same across all gestational ages of both infants.

The main limitation of this approach is that it requires an older sibling to make the prediction. In the absence of a sibling, other models have been proposed to predict birth weight, such as measures based on maternal characteristics [29,30] or using the mother's own birth weight [12]. Our proposed approach requires gestational age of both children and, since gestational age is prone to misclassification, this may be a source of additional error. Even so, including gestational age of both children slightly improved the fit of the predicted birth weight, especially if the first-born child had a low birth weight.

When the birth weight of the first baby is extremely small or extremely high, due to either measurement error or natural variability, this will lead to attenuation of the relation between the birth weights of the two siblings. While this may reduce the ability of the model to predict birth weight, gestational age and birth weight of the older child still explained a large fraction (48%) of the total variation of the birth weight of the younger child.

### Birth weight ratio and mortality

When we examined the risk of neonatal death of the second baby as a function of how close the achieved birth weight was to the predicted birth weight (birth weight ratio), we observed an inverse J-shaped curve for all strata of achieved birth weight above 2000 grams; overall, mortality was higher for infants with a low birth weight ratio, lower for infants who achieved their predicted birth weight, and slightly higher for infants who achieved a birth weight higher than predicted. However, a low achieved birth weight was more strongly associated with a high risk of mortality, even among babies who fulfilled their prediction, and after adjustment for gestational age, which illustrates the limitations in making predictions in the absence of clinical data. Our results indicate that babies within the same category of birth weight have different risks of mortality, as previously suggested [31], depending in part on how well the achieved birth weight agrees with the predicted birth weight. Although the power was considerable reduced, the results were virtually unchanged after restricting the analysis to second-born children born at term whose older sibling survived at least to their 1^st ^birthday.

Our findings showed that a previous child allows a more individualized prediction of birth weight than gestational age alone. The second baby may differ genetically from the older sibling, but it has been suggested that maternal characteristics correlate with birth weight more closely than fetal genes [32]. Whether the first-born child is a valid representative of the growth potential for later children of the same mother may be questionable. If the first baby was growth-restricted, then the birth weight predicted for the second baby will be off the mark to an extent depending on the first-born's degree of growth restriction. Basso et al [33] showed that a rare confounder that strongly decreases birth weight and increases mortality could, at least in theory, explain the whole association between low birth weight and mortality. If a factor with these characteristics exists, and if it has a tendency to recur within the same mother, this would contribute to explaining why even term babies who fulfil their predicted birth weight have a high mortality if their achieved birth weight is low. This could also occur with less severe – and well known-determinants of growth restriction, such as smoking, if they are present in both pregnancies. Babies of mothers with these characteristics will have a "wrong" predicted birth weight and be at higher risk of death even when babies fulfil their prediction. This will be a problem with any time-stable exposure or condition that decreases – or increases – birth weight and increases the risk of neonatal death or of other health problems.

Our study spans a long time period, during which important improvements in the care of premature newborns have occurred. The relatively small absolute number of deaths in our study did not permit analyses of separate time periods. However, adjusting for year of birth did not change our estimates. Our results only refer to neonatal mortality in singleton live births. Results for post-neonatal mortality may be different. Our study is further limited by the lack of information on congenital anomalies or infections, as these conditions increase the risk of both growth restriction and neonatal death. This will limit the predictive value of our estimates, especially at the lower weights. As these conditions may be present in both pregnancies, this mechanism may, in part, explain the strong predictive risk of a small achieved birth weight among babies who appeared to have fulfilled their growth potential. Similarly, we did not have information on maternal morbidity in either pregnancy, and the mother's health status is likely to play an important role in growth.

The main utility of the proposed approach lies, however, in its ability to provide a framework for assessing risk among babies with apparently "normal" birth weight. If individuals whose fetal growth was compromised have an increased risk for adverse health conditions, early identification may improve their outcome through monitoring or intervention. In the presence of detailed clinical information, the proposed method is unlikely to help physicians assess individual risk. However, a discrepancy in birth weight between siblings may constitute a warning sign even in the absence of obvious pathology, and such babies may benefit from increased surveillance. Babies who failed to achieve their predicted birth weight but were classified as "normal" by the regular criterion of small-for-gestation appeared to be at higher risk of delayed motor development [16]. It is thus of interest to explore whether this applies to other outcomes as well, especially among researchers interested in assessing the medium- and long-term effects of impaired fetal growth.

## Conclusion

Our results lend further credibility to the notion that impaired fetal growth is a marker of compromised development. While a low achieved birth weight was a stronger predictor of mortality, a failure to achieve the predicted birth weight was associated with increased mortality at virtually all birth weights. The approach described here may aid clinicians in the identification of babies at higher risk among those with a birth weight in the normal range. It should also be considered as a tool in epidemiologic studies that aim at studying medium- and long-term consequences of fetal growth disruptions.

## Competing interests

The author(s) declare that they have no competing interests.

## Authors' contributions

All authors contributed to the idea and conception of the study. CBP and OB suggested the initial design of the study. All authors contributed to the final design used. CBP analysed the data. All authors contributed to the interpretation of data. CBP and OB drafted the manuscript. All authors revised the manuscript critically for important intellectual content. All authors read and approved the final manuscript.

## Pre-publication history

The pre-publication history for this paper can be accessed here:


